# Diphtheria and Sanitation

**Published:** 1897-09-25

**Authors:** 


### DIPHTHERIA AND SANITATION.

Some very interesting maps were shown at Montreal, on which all the cases of diphtheria which had occurred in New York during the past four years were plotted out house by house. The cases in each year were distinguished by different marks, so that it was possible not only to see in which houses most cases occurred but how they were distributed over the four years in question. The results are most important, showing as they do how largely the incidence of the disease falls upon certain houses, and how many are left untouched. Perhaps more important still is the demonstration of the frequency with which the same house is attacked year after year. Many causes which have nothing to do with the structural fitness of a building for human habitation may of course contribute to the production of a severe outbreak of diphtheria. Of such causes overcrowding is perhaps the most influential, as we should expect to be the case in so infectious a disease. Knowing how the poor live, it is not surprising that when one such case occurs two or three others are apt to follow in quick succession. But the occurrence of cases year after year in the same house tells another tale, and when we note the frequency with which this takes place we cannot but feel that in too many cases the house is at fault. Accepting to the full the statement that the actual essential cause of an attack of diphtheria is an infection by the diphtheria bacillus, we cannot shut our eyes to the fact that, if it is at school that the infection is received, it is in the home that that difference between child and child is produced which makes one who is exposed to infection suffer, and another not. We have been told of various outbreaks of the disease, the incidence of which has fallen especially on certain schools and on certain classes in these schools, and we may admit these as illustrating, and even as proving the existence of a school distribution of infection; but when, on the other hand, we see how often it happens that many cases are marked against one house—not in one group, but in repeated outbreaks year after year—we are driven to the conclusion that although there may be a school distribution of infection there is also a house distribution of a tendency to catch disease; which after all is equally important.

It has been the fashion of late to deny the influence of insanitation in the production of diphtheria, the argument being that while the general sanitation of the country has been improving on all hands during the past 20 years, and while the diseases most definitely connected with sanitary deficiencies have been diminishing, diphtheria has increased on every side. If this stood by itself it might be a valid contention. But it must be remembered that if the increase of diphtheria during recent years is the result of the intrusion of a new influence, viz., the Education Acts, into the problem, as is maintained, that influence may well have overridden and obliterated any tendency to diminution in the prevalence of diphtheria which may have been produced by improved sanitation. The argument, then, entirely falls to the ground, and we think that the results of the inquiry made in New York by Dr. Hermann Biggs will do much to strengthen the old belief that besides infection there is a "house condition" which has a large say in determining who shall suffer. If this is so, something more than the formal "disinfection" now in vogue should be done to the infected, or shall we say the infecting, house. Repeated outbreaks of infectious disease in a given house must be taken as *primâ facie* evidence against its sanitary condition.

